# Analyses of 123 Peripheral Human Immune Cell Subsets: Defining Differences with Age and between Healthy Donors and Cancer Patients Not Detected in Analysis of Standard Immune Cell Types

**DOI:** 10.5772/62322

**Published:** 2016-03-10

**Authors:** Lauren M. Lepone, Renee N. Donahue, Italia Grenga, Simon Metenou, Jacob Richards, Christopher R. Heery, Ravi A. Madan, James L. Gulley, Jeffrey Schlom

**Affiliations:** 1 Laboratory of Tumor Immunology and Biology, Center for Cancer Research, National Cancer Institute, National Institutes of Health, Bethesda, MD, USA; 2 Genitourinary Malignancies Branch, Center for Cancer Research, National Cancer Institute, National Institutes of Health, Bethesda, MD, USA

**Keywords:** Peripheral Blood Mononuclear Cells, Multicolour Flow Cytometry, Cancer, Age

## Abstract

Recent advances in human immunology have led to the identification of novel immune cell subsets and the biological function of many of these subsets has now been identified. The recent US Food and Drug Administration approval of several immunotherapeutics for the treatment of a variety of cancer types and the results of ongoing immunotherapy clinical studies requires a more thorough interrogation of the immune system. We report here the use of flow cytometry-based analyses to identify 123 immune cell subsets of peripheral blood mononuclear cells. The use of these panels defines multiple differences in younger (< 40 years) vs. older (≥ 40 years) individuals and between aged-matched apparently healthy individuals and metastatic cancer patients, aspects not seen in the analysis of the following standard immune cell types: CD8, CD4, natural killer, natural killer-T, regulatory T, myeloid derived suppressor cells, conventional dendritic cells (DCs), plasmacytoid DCs and B cells. The use of these panels identifying 123 immune cell subsets may aid in the identification of patients who may benefit from immunotherapy, either prior to therapy or early in the immunotherapeutic regimen, for the treatment of cancer or other chronic or infectious diseases.

## 1. Introduction

With the recent US Food and Drug Administration approvals of immunotherapeutics such as the checkpoint inhibitor anti-cytotoxic T lymphocyte-associated protein-4 (CTLA-4) and anti-programmed cell death-1 (PD-1)/programmed cell death ligand-1 (PD-L1) monoclonal antibodies (MAbs), and the sipuleucel-T prostate cancer vaccine, as well as results emerging from ongoing clinical studies with other immunotherapeutics, immunotherapy is emerging as a modality for many cancer types and stages. It has long been believed that an individual's immune system can play a role in both the development and control of cancer. It is known that the tumour itself can produce a spectrum of molecules such as immunomodulating cytokines, which can alter the patient's immune system. [1] Moreover, the incidence rates for many cancers increase with age and prior studies have shown that the immune system in older individuals (usually ≥ 65 years) differs from that of younger individuals. [2, 3]

Most prior studies have evaluated changes in an individual's immune system by quantifying so-called “standard parental immune cell types” in the periphery such as CD4, CD8, regulatory T cells (Tregs), B cells, natural killer (NK) cells, NK-T cells, conventional dendritic cells (cDCs) and plasmacytoid DCs (pDCs), and myeloid derived suppressor cells (MDSCs). Recent advances in cellular immunology have identified numerous subsets within each of the above immune cell types via the identification of new markers on immune cells and the use of polychromatic flow cytometry; prior studies have also identified the function(s) of many of these immune cell subsets. [4, 5]

In the studies reported here, we employed MAbs directed against immune cell markers and multi-laser flow cytometry analyses to identify 123 unique immune cell subsets in the peripheral blood of individuals. Since the incidence of cancer rises sharply at the age of 40 and more cancers are being seen recently in younger individuals [6, 7], we have investigated whether changes exist in peripheral immune cell subsets between apparently healthy individuals < 40 and those ≥ 40 years of age; several immune cell subsets were found to be statistically different between these two age groups. We also found that numerous peripheral immune cell subsets can be differentiated between age-matched healthy individuals and patients with a range of advanced human carcinomas. Moreover, we have identified differences among immune cell subsets expressing molecules on their surface that are the targets of existing checkpoint inhibitor therapies such as CTLA-4, PD-1 and PD-L1, as well as other potential targets for which immunotherapies are being developed. To further investigate the differences in peripheral immune cell subsets between healthy donors and advanced cancer patients, microarray analysis was performed on an additional small cohort of healthy donors and cancer patients. While gene pathway analysis demonstrated the regulation of multiple pathways previously associated with cancer, several genes implicated in the regulation of immune cells, including MDSCs and B cells, were also identified as differentially expressed between advanced cancer patients and healthy donors, corroborating the flow cytometry analysis.

## 2. Materials and Methods

### 2.1 Healthy donors and cancer patients

The flow cytometry analysis included 11 healthy donors under the age of 40, 15 healthy donors over the age of 40 and 30 patients with a variety of metastatic solid tumours over the age of 40. The median age of the healthy donor group < age 40 was 26 (range 18–31), with six males and five females. The median age of the healthy donor group > age 40 was 56 (range 46–78), with 12 males and three females. Peripheral blood mononuclear cells (PBMCs) from the healthy donors were obtained from the NIH Clinical Center Blood Bank (NCT00001846), as previously described. [8] The median age of the cancer patients was 64 (range 42–77), with 17 males and 13 females. The cancer patients all had metastatic solid tumours and were enrolled in a Phase I clinical trial (NCT01772004), with PBMCs that were examined in this study obtained prior to the initiation of therapy. The National Cancer Institute Institutional Review Board approved the trial procedures and informed consent was obtained in accordance with the Declaration of Helsinki. Patients had 13 different types of cancer including adrenocortical (n=2), breast (n=3), chordoma (n=1), gastrointestinal (GI) (n=6), lung (n=1), medullary thyroid (n=1), mesothelioma (n=3), neuroendocrine (n=1), ovarian (n=1), pancreatic (n=6), prostate (n=1), renal cell (n=3) and spindle cell (n=1) cancer, and the median number of prior anti-cancer therapies was 3 (range 1–15).

In an additional cohort of samples assessed by microarray, PBMCs were obtained from five healthy donors (median age: 54, three male, two female) from the NIH Clinical Center Blood Bank, as well as four patients with advanced GI cancer (median age: 56.5, two male, two female) enrolled in a Phase I study at the NCI (NCT00088413) [9]; PBMCs used in this study were isolated prior to treatment.

### 2.2 Antibodies and flow cytometry

**Figure 1.**
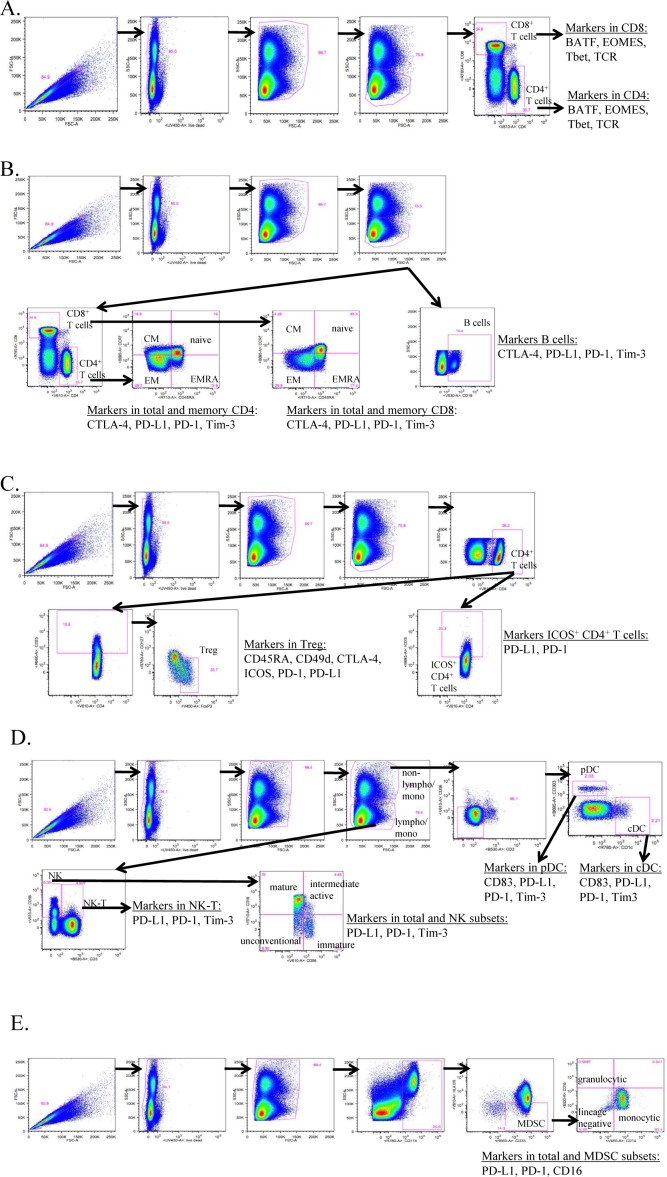
Gating strategy of five staining panels to identity 123 peripheral immune cell subsets by flow cytometry. One vial of frozen peripheral blood mononuclear cells (PBMCs) per subject was thawed and then stained using 30 unique markers in five separate immune flow cytometry panels to identify a total of 123 peripheral immune cell subsets. Each panel contained up to 11 markers. Panel 1 (A) identified markers in CD8^+^ and CD4^+^ T cells involved in PD-1 signalling. Panel 2 (B) identified the parental cell types of CD4^+^ and CD8^+^ T cells and B cells, as well as markers involved in function and maturation. Panel 3 (C) identified ICOS^+^ CD4^+^ T cells and the parental cell type of Tregs, as well as markers involved in function. Panel 4 (D) identified the parental cell types of NK, NK-T, cDC and pDC, as well as markers involved in maturation and function. Panel 5 (E) identified the parental cell type of MDSC, as well as markers involved in maturation and function. Samples were collected on an LSR II flow cytometer equipped with UV, red, blue and violet lasers, and analysed using FlowJo, with gating set using fluorescence minus one controls.

Multicolour flow cytometry was performed on frozen PBMCs as previously described. [10] One vial of PBMCs was thawed per cancer patient or healthy donor. Cells were counted and plated in five different wells per subject in a 96-well plate with one million PBMCs per well. Each of the five wells was used to stain a different panel with a maximum of 11 colours per panel in each well. Staining was performed using five panels (Supplemental Table 1) to identify markers involved in PD-1 signalling (panel 1) and in subsets of CD4^+^ T cells, CD8^+^ T cells and B cells (panel 2), Tregs (panel 3), NKs, NK-T, cDCs and pDCs (panel 4), and MDSCs (panel 5). Using the outlined gating strategy (Figure 1), these five staining panels with up to 11 antibodies per panel identified a total of 123 peripheral immune cell subsets (Supplemental Table 2), which included nine parental immune cell types and 114 subsets related to maturation and function within the parental types. Optimal amounts of antibodies for staining were determined by titration experiments. Briefly, one million PBMCs per test were incubated for 15 minutes at 4°C with 2 μL of human TruStain FcX (Biolegend, San Diego, CA) and Live Dead Fixable Stain Blue (Invitrogen, Waltham, MA). Surface antibodies were added for 30 minutes at 4° C. Cells were then washed, fixed and permeabilized using the Intracellular Fixation and Permeabilization Buffer Set (eBioscience, San Diego, CA) and stained with intracellular antibodies for 30 minutes at room temperature. Samples were acquired on a BD LSRII flow cytometer (BD Biosciences, San Jose, CA) equipped with four lasers (UV, violet, blue and red; configuration and filter sets of LSR II listed in Supplemental Figure 1) and analysed using FlowJo V9.7 for Macintosh (Treestar, Ashland, OR). The gating strategy identified 123 peripheral immune cell subsets from the five staining panels that each contained up to 11 markers per panel (Figure 1), with non-viable cells excluded and negative gates set based on fluorescence minus one controls. All values were reported as a percentage (%) of PBMCs in order to help eliminate the bias that could occur in the smaller populations with fluctuations in leukocyte subpopulations. [11]

### 2.3 Microarray

Total RNA was isolated from PBMCs using the Qiagen RNAeasy Plus minikit (Valencia, CA) according to the manufacturer's instructions and quality-checked on an Agilent Bioanalyzer (Santa Clara, CA). All samples used for microarray analysis had an RNA integrity number >9; 100 ng of RNA was reverse transcribed and amplified using an Ambion WT expression kit (Austin, TX), following the manufacturer's suggested protocols. Sense strand cDNA was fragmented and labelled using an Affymetrix WT terminal labelling kit (Santa Clara, CA). Four replicates of each group were hybridized to Affymetrix human Gene ST 2.0 GeneChip in an Affymetrix hybridization oven at 45°C, at 60rpm for 16 hours. Washing and staining were performed on an Affymetrix Fluidics Station 450 and scanned on an Affymetrix GeneChip Scanner 3000. Data were collected using Affymetrix AGCC software.

### 2.4 Statistic

Statistical analyses for flow cytometry data were performed using GraphPad Prism 6 (GraphPad Software, La Jolla, CA). All p-values were calculated using the Mann-Whitney test. In view of the large number of tests performed, p-values were adjusted using Holm's method (step-down Bonferroni) to account for the increased probability of Type I errors (false positive) that occur when multiple outcome measures are assessed. [12] Adjustment was made for the number of subsets with a frequency above 0.01% of PBMCs (n=9 for standard subsets, n=29 for subsets in CD4^+^ T cells, n=25 for CD8^+^ T cells, n=5 for Tregs, n=14 for NK cells, n=3 for NK-T cells, n=4 for B cells, n=2 for cDCs, n=3 for pDCs and n=15 for MDSCs). Subsets with a potentially biologically relevant change were defined as subsets with an adjusted p<0.05, the median of groups showing a > 50% difference, and a frequency > 0.01% of PBMCs.

Statistical and clustering analysis for the microarray experiment was performed with Partek Genomics Suite software (St. Louis, MO) and employing a RMA normalization algorithm. Differentially expressed genes were identified via ANOVA analysis. Genes that were up- or down-regulated more than 1.5-fold and with a p<0.05 were considered significant. Significant genes were analysed for the enrichment for pathways using Ingenuity Pathway Analysis software (Redwood City, CA).

## 3. Results

### 3.1 Differences in peripheral immune cell subsets with age in healthy donors

**Figure 2.**
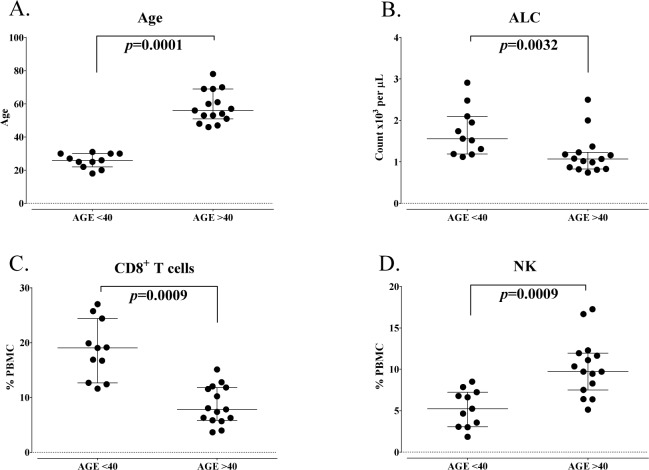
Standard parental immune cell types in healthy donors under and over the age of 40. A: Healthy donors included in this analysis were separated as younger (age less than 40 years, n=11) and older (age greater than 40 years, n=15). B: Absolute lymphocyte count (ALC) of healthy donors under and over age 40. Graphs display median age or ALC with 25–75 percentiles. P-value was calculated using the Mann-Whitney test. CD8^+^ T cells (C) and natural killer (NK) cells (D) were different between healthy donors under and over age 40, as defined by an adjusted p<0.05, the median of groups showing a > 50% difference, and a frequency above 0.01% of peripheral blood mononuclear cells (PBMCs). Graphs display median frequency as a percentage of PBMCs with 25–75 percentiles. P-value was calculated using the Mann-Whitney test and with Holm adjustment made for multiple comparisons using the number of standard immune cell types with a frequency above 0.01% of PBMCs (n=9).

Using multiparameter flow cytometry, a total of 123 peripheral immune cell subsets were examined, which included the nine standard parental immune cell types – CD4^+^ and CD8^+^ T cells, Tregs, B cells, NK and NK-T cells, cDCs, pDCs and MDSCs – and 114 subsets of these cell types relating to maturation and function (Table 1). As the risk of cancer rises at age 40 [6, 7], donors were separated into younger and older groups, using this age as a cut-off. PBMCs were assayed from 11 healthy donors under the age of 40 and 15 healthy donors over the age of 40 (Figure 2A). Compared to the younger group, the older group had a significantly lower absolute lymphocyte count (ALC) (p=0.0032, Figure 2B). No statistical differences were evident when subsets were examined by age decade within the groups or by gender (data not shown).

**Table 1.** Flow cytometry analysis of parental immune cell types in PBMCs using 30 unique markers to identify 123 subsets

**CD4^+^ T cells:** Helper T lymphocytes (32 subsets) **CD8^+^ T cells:** Cytotoxic T lymphocytes (29 subsets) **Markers of PD-1 pathway and T cell activation (in CD4 and CD8):** – **EOMES:** activation – **TCR:** activation – **Tbet:** activation – **BATF:** activation/exhaustion **Maturation status of T cells (in CD4 and CD8):** – **Naïve:** CD45RA^+^ CCR7^+^ – **Central Memory (CM):** CD45RA^−^ CCR7^+^ – **Effector Memory (EM):** CD45RA^−^ CCR7^−^ – **Terminal (EMRA):** CD45RA^+^ CCR7^−^ **T cell markers (in CD4 and CD8):** – **CTLA-4:** inhibition – **PD-1:** activation/inhibition – **PD-L1:** activation/cross-inhibition – **Tim-3:** inhibition – **ICOS:** activation (only on CD4) **Tregs:** Regulatory T lymphocytes (CD4^+^ CD25^+^ FoxP3^+^ CD127^−^) (7 subsets) – **CD45RA:** Tregs highly expandable in vitro – **CTLA-4:** Treg suppression – **CD49d**^−^: suppressive Tregs – **ICOS:** Treg suppression – **PD-1:** activation/inhibition – **PD-L1:** cross-inhibition **B cells:** CD19^+^ (5 subsets) – **CTLA-4:** inhibition – **Tim-3:** inhibition – **PD-1:** activation/inhibition – **PD-L1:** cross-inhibition **NK:** Natural killer cells (CD56^+^ CD3^−^) (20 subsets) – **CD16^+^ CD56^dim^:** Mature, lytic – **CD16^+^ CD56^br^:** Functional intermediate, lytic and cytokine production – **CD16^−^ CD56^br^:** Immature, cytokine production, abundant in placenta – **CD16^−^ CD56^dim^:** non-lytic, non-cytokine production – **Tim-3:** activation – **PD-1:** activation/inhibition – **PD-L1:** cross-inhibition **NK-T:** CD56^+^ CD3^+^ (4 subsets) – **Tim-3:** activation – **PD-1:** activation/inhibition – **PD-L1:** cross-inhibition **cDCs:** conventional dendritic cells (DCs) (CD3^−^CD56^−^CD1c^+^CD303^−^) (5 subsets) **pDCs:** plasmacytoid DCs (CD3^−^CD56^−^CD1c^−^CD303^+^) (5 subsets) **Markers of DC activation** – **CD83:** activation – **Tim-3:** inhibition – **PD-1:** activation/inhibition – **PD-L1:** cross-inhibition **MDSCs:** Myeloid derived suppressor cells (CD11b^+^ HLA-DR^low/-^CD33^+^) (16 subsets) – **CD14:** common myeloid marker – **CD15:** granulocyte marker – **CD16:** immature MDSCs – **PD-1:** activation/inhibition – **PD-L1:** cross-inhibition

Frozen PBMCs were thawed then stained using 30 unique markers in 5 immune flow cytometry panels to identify a total of 123 peripheral immune cell subsets. Samples were collected on an LSR II flow cytometer equipped with U V, red, blue, and violet lasers, and analyzed using FlowJo with gating set using fluorescence minus one controls. Nine standard immune cell types as well as 114 additional subsets relating to maturation and function were compared between healthy donors under and over the age of 40, and between patients with metastatic cancer and age-matched healthy donors. ——–

BATF, basic leucine zipper transcription factor ATF-like; CTLA-4, cytotoxic T lymphocyte-associated protein-4; EOMES, eomesodermin; FoxP3, forkhead box P3; HLA, human leukocyte antigen; ICOS, inducible T cell co-stimulator; PBMCs, peripheral blood mononuclear cells; PD-1, programmed cell death-1; PD-L1, programmed cell death ligand-1; Tbet, T box expressed in T cells; TCR, T cell receptor; Tim-3, T cell immunoglobulin and mucin domain-3.

Differences in peripheral immune cell subsets were identified as significant and relevant if the adjusted Holm p-value was < 0.05, if medians of the group were at least 50% different and if the subset had a frequency of above 0.01% of PBMCs. Using these criteria, of the nine standard immune cell types, seven were unchanged and two cell types (CD8^+^ T cells, NK cells) were significantly changed with age (Table 2). Healthy donors over the age of 40 had on average 60% less total CD8^+^ T cells compared to those under the age of 40 (median % of PBMCs = 8 in old and 19 in young, p=0.0009, Figure 2C). Older healthy donors, on the other hand, had on average 85% more NK cells compared to younger healthy donors (median % of PBMCs = 10 in older and 5 in younger, p=0.0009, Figure 2D).

**Figure 3.**
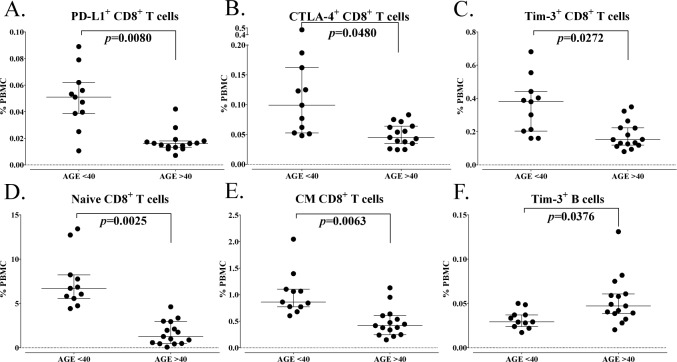
Differences in immune cell subsets related to maturation and function between healthy donors under and over the age of 40. A total of 114 immune cell subsets were analysed relating to maturation and function within the standard subsets. Subsets were considered different if a subset had an adjusted p<0.05, the median of groups showing a > 50% difference, and a frequency above 0.01% for peripheral blood mononuclear cells (PBMCs). A-F: Representative graphs are shown for notable subsets related to activation and maturation, with differences between healthy donors under and over the age of 40 indicated. Graphs display median frequency as a percentage of PBMCs with 25–75 percentiles. P-value was calculated using the Mann-Whitney test, with Holm adjustment made for multiple comparisons using the number of subsets within each standard subset with a frequency above 0.01% of PBMCs (n=29 for CD4^+^ T cells, 25 for CD8^+^ T cells, 5 for Tregs, 14 for natural killer cells (NK), 3 for NK-T cells, 4 for B cells, 2 for conventional dendritic cells (cDCs), 3 for plasmacytoid DCs (pDCs) and 15 for myeloid derived suppressor cells (MDSCs)).

Of the 114 subsets relating to maturation and function within the standard cell types, 12 subsets were significantly different in older healthy donors compared to younger individuals (Table 3A). The most notable decreases in subsets relating to maturation and function in healthy donors over age 40 were evident in CD8^+^ T cell subsets; these included on average 70% fewer PD-L1^+^CD8^+^ T cells (p=0.0080, Figure 3A), 55% fewer CTLA-4^+^CD8^+^ T cells (p=0.0480, Figure 3B) and 60% fewer T cell immunoglobulin and mucin domain-3 (Tim-3)^+^CD8^+^ T cells (p=0.0272, Figure 3C). The markers PD-L1, CTLA-4 and Tim-3 are inhibitory receptors involved in immune checkpoint pathways and are increased upon T cell activation and differentiation. [13] Donors over 40 also had decreases in subsets relating to maturation, with lower frequencies of naïve (p=0.0025, Figure 3D) and central memory (CM) (p=0.0063, Figure 3E) CD8^+^ T cells. The only subset that showed a trend (p=0.038) of increase in healthy donors over age 40 was Tim-3^+^ B cells (Figure 3F). Tim-3 is an inhibitory marker often examined in T cells and NK cells, and only rarely described in minor populations of B cells [14]. Total B cells were unchanged.

**Table 2.** Standard parental immune cell types in healthy donors under and over age 40

Immune cell type	Age <40	Age >40	P value	Direction of age >40
CD8^+^ T cells	19	8	0.0009	↓
NK	5	10	0.0040	↑
CD4^+^ T cells	34	29	0.8975	=
Tregs	1.4	1.5	0.9999	=
B cells	11	16	0.1645	=
NK-T	2.4	1.3	0.4116	=
cDC	0.4	0.3	0.9999	=
pDC	0.2	0.2	0.8975	=
MDSC	5	4	0.9999	=

A total of 9 standard parental immune cell types were analyzed. Values are displayed as median % of PBMCs. P-value was calculated with the Mann Whitney test with Holm adjustment made for multiple comparisons using the number of standard immune cell types with frequency above 0.01% of PBMCs (n=9). Differences were defined as subsets with an adjusted p<0.05, medians at least 50% different, and frequency above 0.01% of PBMCs. ——–

cDC, conventional dendritic cells; MDSC, myeloid derived suppressor cells; NK, natural killer; PBMCs, peripheral blood mononuclear cells; pDC, plasmacytoid DC; Tregs, regulatory T cells.

Other immune cell subsets also showed trends in differences with age; trends were defined as those subsets having an unadjusted p-value < 0.01 (without a significant Holm adjusted p-value), as well as at least a 50% difference in medians and a frequency of PBMCs above 0.01%. Using these criteria, six additional subsets were found to have trends in differences with age (Table 3B). While total CD4^+^ T cells did not change, the most notable trends were evident in several CD4^+^ T cell subsets expressing PD-1, which is an inhibitory receptor that is increased upon T cell activation [13]. Healthy donors over the age of 40 had higher levels of PD-1 in total CD4^+^ T cells, inducible T cell co-stimulator (ICOS)^+^CD4^+^ T cells, effector memory (EM) CD4^+^ T cells and CM CD4^+^ T cells (Table 3B). Thus, healthy donors over age 40 had lower levels of activation markers on CD8^+^ T cells, varied maturation status of CD8^+^ T cells and trends of higher activation markers in CD4^+^ T cells compared to healthy donors under age 40.

### 3.2 *Differences in peripheral immune cell subsets between patients with carcinoma and age-matched healthy donors*

**Figure 4.**
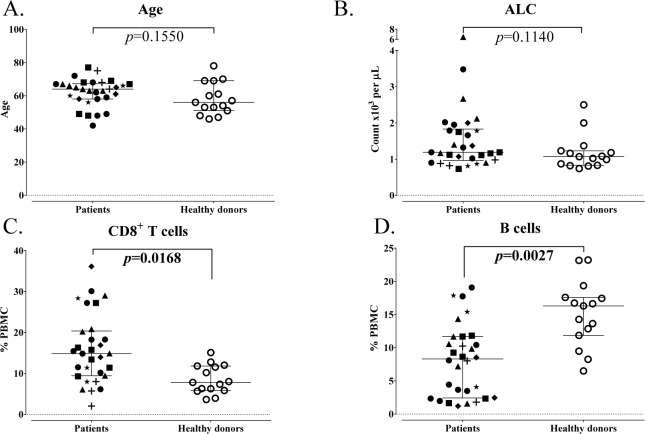
Standard parental immune cell types in age-matched advanced cancer patients and healthy donors. A: Patients with advanced cancer (n=30) and healthy donors (n=15) included in this analysis were age-matched above age 40. B: Absolute lymphocyte count (ALC) of cancer patients and healthy donors. Graphs display median age or ALC with 25–75 percentiles; cancer type indicated by shape. P-value was calculated using the Mann-Whitney test. CD8^+^ T cells (C) and B cells (D) were different in cancer patients and healthy donors, as defined by an adjusted p<0.05, the median of groups showing a > 50% difference, and a frequency above 0.01% for peripheral blood mononuclear cells (PBMCs). Graphs display median frequency as a percentage of PBMCs with 25–75 percentiles; cancer type indicated by shape (square: GI (anal, colon, oesophageal); n=6; triangle: pancreatic, n=6; star: breast, n=3; plus sign: mesothelioma, n=3; diamond: renal cell, n=3; closed circle: other (adrenocortical, chordoma, lung, medullary thyroid, neuroendocrine, ovarian, prostate, spindle cell), n=9; open circle: healthy donors, n=15). P-value was calculated using the Mann-Whitney test, with Holm adjustment made for multiple comparisons using the number of standard subsets with a frequency above 0.01% for PBMCs (n=9).

PBMCs were evaluated for 30 patients with different types of metastatic solid tumours and 15 age-matched healthy donors, all over the age of 40 (p=0.1550, Figure 4A). The ALC was similar in both groups (p=0.1140, Figure 4B). Using the same criteria as described in the age analysis, seven of the standard parental immune cell types were similar and two (CD8^+^ T cells, B cells) were significantly different between cancer patients and healthy donors (Table 4). Surprisingly, cancer patients had on average 90% more CD8^+^ T cells compared to age-matched healthy donors (median % of PBMCs = 15 in patients and 8 in healthy donors, p=0.0168, Figure 4C). On the other hand, cancer patients also had on average 50% less total B cells compared to healthy donors (median % of PBMCs = 8 in patients and 16 in healthy donors, p=0.0027, Figure 4D). No notable differences were evident when subsets were compared between patients with different cancer types; however, this was likely due to the small number of patients within each indication that were examined (data not shown).

**Figure 5.**
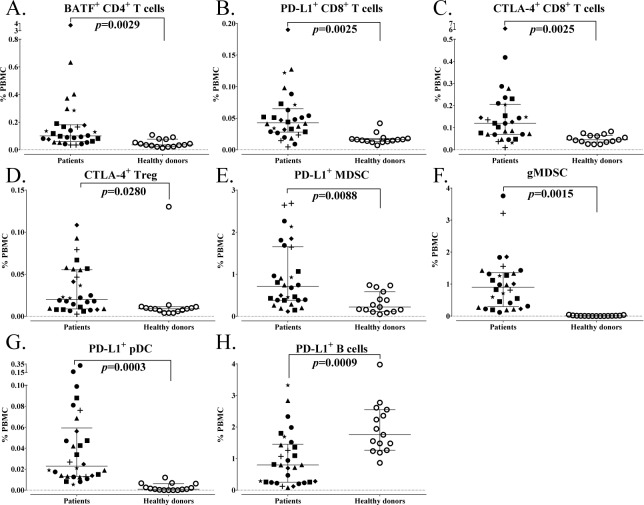
Differences in immune cell subsets related to maturation and function between age-matched advanced cancer patients and healthy donors. A total of 114 subsets were analysed related to maturation and function within the standard subsets. Subsets were considered different if a subset had an adjusted p<0.05, the median of groups showing a > 50% difference, and a frequency above 0.01% for peripheral blood mononuclear cells (PBMCs). A-H: Representative graphs are shown for notable subsets with differences between cancer patients and healthy donors. Graphs display median frequency as a percentage of PBMCs with 25–75 percentiles; cancer type indicated by shape (square: GI (anal, colon, oesophageal), n=6; triangle: pancreatic, n=6; star: breast, n=3; plus sign: mesothelioma, n=3; diamond: renal cell, n=3; closed circle: other (adrenocortical, chordoma, lung, medullary thyroid, neuroendocrine, ovarian, prostate, spindle cell), n=9; open circle: healthy donors, n=15). P-value was calculated using the Mann-Whitney test, with Holm adjustment made for multiple comparisons using the number of subsets within each standard subset with a frequency above 0.01% for PBMCs (n=29 for CD4^+^ T cells, 25 for CD8^+^ T cells, 5 for Tregs, 14 for natural killer (NK) cells, 3 for NK-T cells, 4 for B cells, 2 for conventional dendritic cells (cDCs), 3 for plasmacytoid DCs (pDCs) and 15 for myeloid derived suppressor cells (MDSCs)).

Of the 114 subsets related to maturation and function within the standard immune cell types, 23 were changed with cancer (Table 5A). Some of the notable increases seen in cancer patients included subsets of T cells expressing basic leucine zipper transcription factor, ATF-like (BATF), PD-L1 and CTLA-4, which are inhibitory markers involved in immune checkpoint pathways and are increased upon T cell activation [13, 15]. While the percentage of CD4^+^ T cells was similar between cancer patients and healthy donors, cancer patients had 180% higher levels of BATF^+^CD4^+^ T cells (p=0.0029, Figure 5A). Along with an increase in the percentage of CD8^+^ T cells, cancer patients also had 170% higher levels of PD-L1^+^CD8^+^ T cells (p=0.0025, Figure 5B) and 165% higher levels of CTLA-4^+^CD8^+^ T cells (p=0.0025, Figure 5C). Using the less stringent criteria as described above in the age analysis to identify subsets with trends, cancer patients showed increases in T box expressed in T cells (Tbet^+^)CD8^+^ and eomesodermin (EOMES)^+^CD8^+^, as well as Tbet^+^CD4^+^ cells (Table 5B). These subtypes are known to be increased upon activation and differentiation. [16]

**Table 3.** Differences in immune cell subsets relating to maturation and function between healthy donors under and over age 40

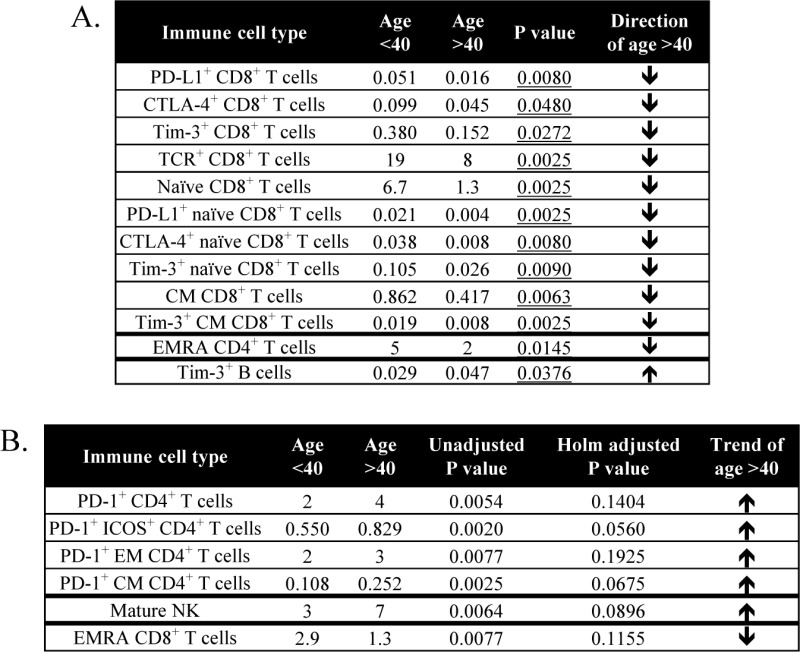

A total of 114 subsets were analyzed relating to maturation and function within the standard subsets. A: Table displays subsets that were different in healthy donors under and over age 40, as defined by an adjusted p<0.05, medians at least 50% different, and frequency above 0.01% of PBMCs. Values represent median % of PBMCs, and p-value was calculated with the Mann Whitney test with Holm adjustment made for multiple comparisons using the number of subsets within each standard subset with frequency above 0.01% of PBMCs (n=29 for CD4^+^ T cells, 25 for CD8^+^ T cells, 5 for Tregs, 14 for NK cells, 3 for NK-T cells, 4 for B cells, 2 for cDCs, 3 for pDCs, 15 for MDSCs). B: Table displays subsets with notable trends between healthy donors under and over age 40, as defined by unadjusted p< 0.01, medians at least 50% different, and frequency above 0.01% of PBMCs. Values represent median % of PBMCs.

——–

cDC, conventional dendritic cells; CM, central memory; CTLA-4, cytotoxic T lymphocyte-associated protein-4; EM, effector memory; EMRA, terminally differentiated effector memory; ICOS, inducible T cell co-stimulator; MDSC, myeloid derived suppressor cells; NK, natural killer; PBMCs, peripheral blood mononuclear cells; pDC, plasmacytoid DC; PD-1, programmed cell death-1; PD-L1, programmed cell death ligand-1; TCR, T cell receptor; Tim-3, T cell immunoglobulin and mucin domain-3; Tregs, regulatory T cells.

Cancer patients also had notable increases in subsets involving maturation and function in suppressive immune cells. While the percentage of Tregs and MDSCs was similar between cancer patients and healthy donors, cancer patients had higher levels of a Treg subset that expressed CTLA-4 (p=0.0280, Figure 5D), which is a suppressive marker for these regulatory cells [17], as well as increased levels of PD-L1^+^ MDSCs (p=0.0088, Figure 5E), a marker that has been implicated in suppressive function [18]. Cancer patients also had higher levels of granulocytic MDSCs (gMDSCs) (p=0.0015, Figure 5F), which have been found to be more suppressive than monocytic MDSCs (mMDSCs). [19]

While the percentage of pDC levels was also similar between cancer patients and healthy donors, cancer patients also had higher levels of PD-L1^+^ pDCs (p=0.0003, Figure 5G), which can have immunosuppressive and tolerogenic properties [20]. Cancer patients, on the other hand, had lower levels of PD-L1^+^ B cells (p=0.0009, Figure 5H), a marker that is upregulated upon B cell activation [21]. Thus, patients with advanced cancer had elevated levels of activation markers involved in immune checkpoint pathways in both CD4^+^ and CD8^+^ T cells, higher Tregs and MDSCs with a suppressive phenotype, and differences in PD-L1 expression on antigen-presenting cells (APCs) compared to age-matched healthy donors.

### 3.3. *Differences in gene expression profiles between cancer patients and age-matched healthy donors*

**Figure 6.**
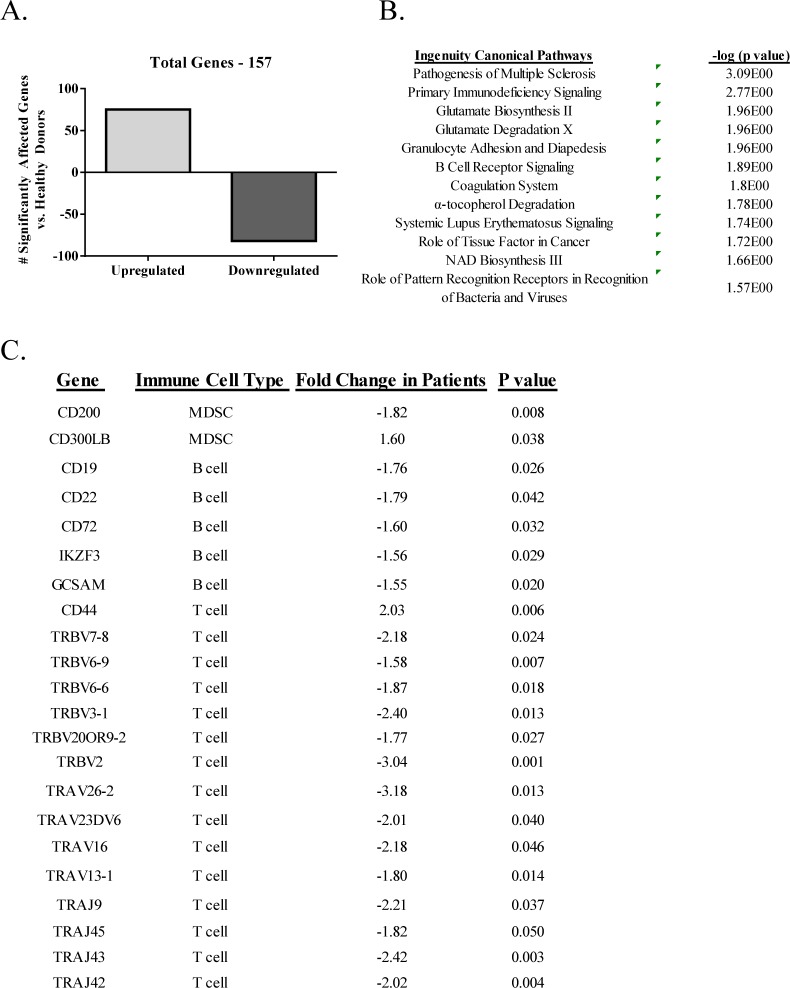
Microarray analysis in PBMCs from metastatic patients with GI cancer and age-matched healthy donors. Microarray analysis was performed on RNA isolated from PBMCs of patients with advanced GI cancer (n=4) and age-matched healthy donors (n=5). A: Total number of significantly affected genes (p < 0.05 and fold change > |1.5|). B: Ingenuity pathway analysis of the 157 significantly affected genes. Pathways are listed in the left column and p-value (Fisher's exact test) is listed in the right column. C: Key immune related genes identified as differentially expressed (p < 0.05 and fold change > |1.5|), with known key immunological function between advanced cancer patients and age-matched healthy donors. Includes gene name, known cell type, fold change and p-value.

To determine whether changes in immune cell subsets seen at the protein level could be corroborated at the gene level, a study was performed in which gene expression profiling was performed in PBMCs from patients with advanced GI cancer (n=4) and age-matched healthy donors (n=5). A total of 157 genes were significantly different (p-value <0.05 and fold change > |1.5|) between the two groups (Figure 6A). Pathway analysis demonstrated that genes were localized to several pathways, including B cell receptor signalling and glutamate signalling pathways (Figure 6B). Several genes implicated in the regulation of immune cells including MDSCs, B cells and T cells were identified as differentially expressed between the two groups (Figure 6C). For MDSCs, this included decreases in CD200, a ligand receptor expressed on myeloid cells involved in the inhibition of MDSC signalling [22], as well as increases in CD300LB, an immune receptor expressed in myeloid cells involved in the activation of suppressor function [23]. With regard to B cells, changes included decreases in the gene expression of CD19, CD22, CD72, IKZF3 and GCSAM in cancer patients compared to healthy donors. CD19 is the hallmark differentiation antigen of B cell lineage, positively regulating antigen receptor signalling in these cells [24]; CD22 and CD72 are involved in B cell maturation [25, 26], IKZF3 is a transcription factor involved in B cell proliferation and development [27], and GCSAM, also known as HGAL, has been shown to activate B cell receptor signalling by enhancing its kinase activity [28]. Multiple T cell receptor genes were also decreased in both the alpha and beta families.

**Table 4.** Standard parental immune cell types in age-matched advanced cancer patients and healthy donors

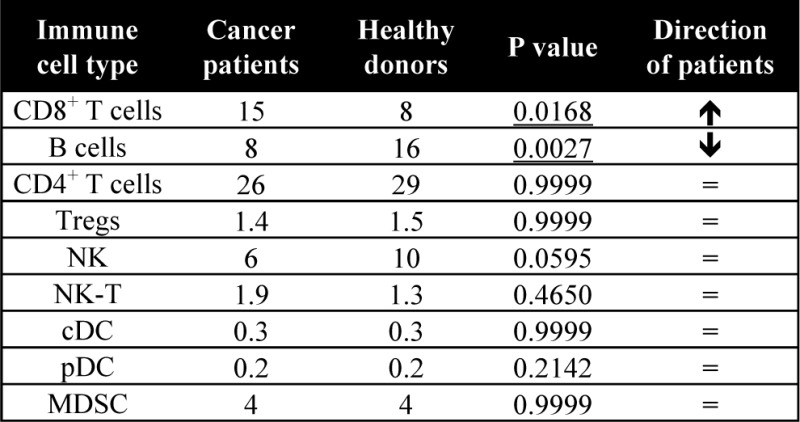

A total of 9 standard parental immune cell types were analyzed. Values are displayed as median % of PBMCs. P-value was calculated with the Mann Whitney test with Holm adjustment made for multiple comparisons using the number of standard immune cell types with frequency above 0.01% of PBMCs (n=9). Differences were defined as subsets with an adjusted p<0.05, medians at least 50% different, and frequency above 0.01% of PBMCs.

——–

cDC, conventional dendritic cells; MDSC, myeloid derived suppressor cells; NK, natural killer; PBMCs, peripheral blood mononuclear cells; pDC, plasmacytoid DC; Tregs, regulatory T cells.

## 4. Discussion

With the expanded use of immunotherapeutics such as checkpoint inhibitor MAbs, vaccines and immune modulators in the management of many cancer types, the identification (either prior to therapy and/or early in the therapeutic regimen) of patients most likely to benefit from immunotherapy becomes more important. For example, evidence has emerged from studies of patients with melanoma and other tumour types of prognostic factors derived from tumour biopsies such as: (a) the number of mutations, (b) the expression of PD-L1 on tumour cells and/or (c) the presence of immune infiltrate. However, using these factors has not always provided a good surrogate of patient benefits with clear outliers (both positive and negative) for all three of these parameters. Moreover, with the exception of melanoma and some metastatic lesions to the skin of other tumour types, biopsies of metastatic lesions of most solid tumours are not always feasible and numerous studies have shown that the phenotype of primary tumours often differs from metastatic lesions. Analyses of immune cells in the periphery may very well aid – along with other parameters mentioned – in selecting patients most likely to benefit from and/or in identifying patients early on in the agent regimen who will benefit from a specific immunotherapy.

**Table 5.** Differences in immune cell subsets relating to maturation and function between age-matched advanced cancer patients and healthy donors

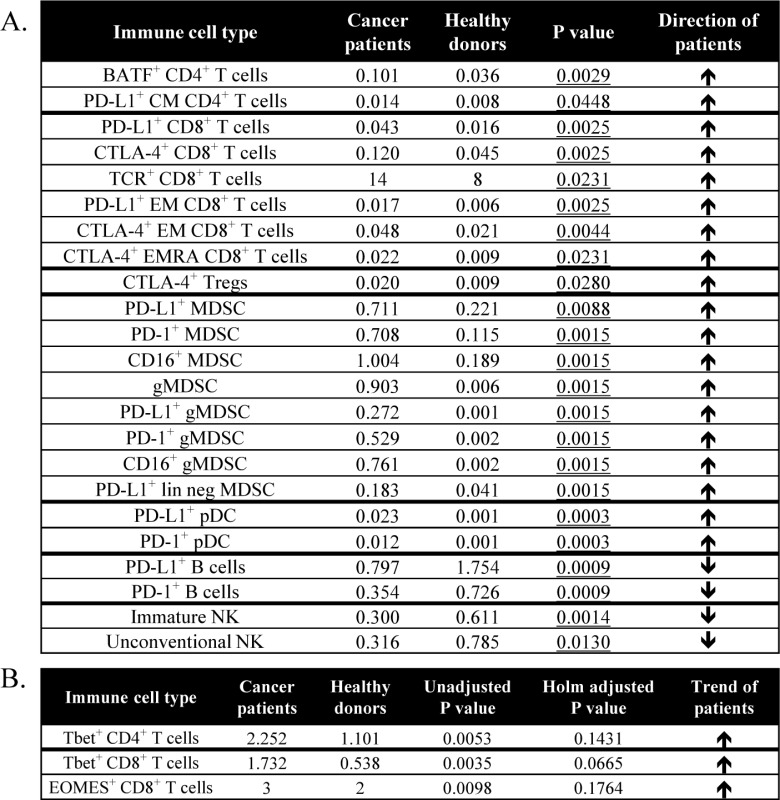

A total of 114 subsets were analyzed relating to maturation and function within the standard subsets. **A:** Table displays subsets that were different in cancer patients and age-matched healthy donors, as defined by an adjusted p<0.05, medians at least 50% different, and frequency above 0.01% of PBMCs. Values represent median % of PBMCs, and p-value was calculated with the Mann Whitney test with Holm adjustment made for multiple comparisons using the number of subsets within each standard subset with frequency above 0.01% of PBMCs (n=29 for CD4^+^ T cells, 25 for CD8^+^ T cells, 5 for Tregs, 14 for NK cells, 3 for NK-T cells, 4 for B cells, 2 for cDCs, 3 for pDCs, 15 for MDSCs). **B:** Table displays subsets with notable trends between cancer patients and healthy donors as defined by unadjusted p< 0.01, medians at least 50% different, and frequency above 0.01% of PBMCs. Values represent median % of PBMCs.

——–

BATF, basic leucine zipper transcription factor ATF-like; cDC, conventional dendritic cells; CM, central memory; CTLA-4, cytotoxic T lymphocyte-associated protein-4; EM, effector memory; EMRA, terminally differentiated effector memory; EOMES, eomesodermin; gMDSCs, granulocytic mononuclear derived suppressor cells; lin neg MDSCs, lineage negative MDSCs; NK, natural killer; PBMCs, peripheral blood mononuclear cells; pDC, plasmacytoid DC; PD-1, programmed cell death-1; PD-L1, programmed cell death ligand-1; Tbet, T box expressed in T cells; TCR, T cell receptor; Tim-3, T cell immunoglobulin and mucin domain-3; Tregs, regulatory T cells.

Recent advances in human immunology using multicolour flow cytometry and high-throughput computation analysis (cytomics) now allow for the identification of numerous individual peripheral immune cell subsets [29–31]; moreover, the biological function and relevance of many of these subsets have been described [5]. To our knowledge, the study reported here is the most comprehensive to date, analysing 123 different peripheral immune cell subsets with a focus on subsets related to maturation and function (Table 1), comparing healthy donors under vs. over the age of 40, and comparing metastatic cancer patients with age-matched healthy donors. It should be noted that all comparisons between groups in this study employed the Mann-Whitney test and resulting p-values were adjusted using the step-down Holm adjustment for multiple comparisons. This adjustment was applied to account for the increased probability of Type I errors (false positives) that occur when multiple outcome measures (e.g., 123 subsets) are assessed. However, as p-value adjustments also increase the risk of Type II errors (false negatives), we additionally performed a less stringent analysis evaluating unadjusted p-values and identified trends of changes in subsets with an unadjusted p<0.01.

Changes in the immune system with aging are evidenced by the observation that older individuals have a higher prevalence of autoimmunity, chronic diseases and cancer [32]. Previous studies examining peripheral immune cells during aging have focused primarily on certain standard immune cell types and, in some cases, on their memory status. In addition, prior studies have examined PBMCs of individuals in a young group, defined as aged 18–34, vs. an older group, above the age of 65 [33, 34]; other studies have also used an older cut-off ranging between 60–65 years of age in order to separate older from younger individuals [35–37].

In the study reported here, age 40 [6, 7] was used as a demarcation to define younger and older healthy donor groups, as the risk of cancer rises substantially at age 40. With more cancers also arising earlier in life, it is also important to define the relationship of age on immune cell subsets evaluating a younger age population, rather than focusing on populations aged 65 and above. In this study, differences were not evident when subsets (albeit smaller number per group) were examined by age decade or gender; however, as described above, a number of changes were identified between healthy donors when age groups were defined as below or above the age of 40.

We show here for the first time that healthy donors over the age of 40 had lower ALC, lower CD8^+^ T cells, lower activation markers within CD8^+^ T cells, lower naïve and central memory CD8^+^ T cells and higher NK cells, compared to those under the age of 40 (Figures 2 and 3). NK results are in agreement with those of others reporting an increase in total NK cells with age; however, this increase of NK cells in older individuals was reported when examining donors according to young and old extremes, and when using age 60 as a cut-off to define older individuals [38–40]. In prior studies, using age 60 as a cut-off for older individuals, it has been shown that the CD8^+^ T cell compartment was more affected by age than CD4^+^ T cells [32, 35]. The most notable changes in the studies reported here with regard to age (under vs. over 40) were evident in the T cell compartment and specifically involved CD8^+^ T cells.

In addition to the standard immune cell types, we also examined many subsets related to maturation and function with known biological importance, including immune cells expressing immune checkpoints such as PD-1, PD-L1, CTLA-4 and Tim-3. With regards to maturation status, it is generally accepted that there is a decrease in naïve T cells and an increase in memory T cells with age [2]. It was shown here that healthy donors > 40 years had a decrease in both naïve and central memory CD8^+^ T cells. This substantiates and extends a prior report that elderly individuals have a decrease in both naïve and central memory CD8^+^ T cells in cases where younger donors with an average age of 30 were compared to older donors with an average age of 70 [35]. It has also been reported that T cell signalling can be altered with aging, involving the expression of T cell receptor (TCR) components, signal transduction and the expression of costimulatory receptors [41]. The most notable differences in our age analysis were related to activation markers in CD8^+^ T cells. We found (Figure 3) that healthy donors over the age of 40 had decreases in CD8^+^ T cells that expressed PD-L1, CTLA-4 and Tim-3, which are inhibitory molecules involved in T cell immune checkpoints and can serve as markers of immune activation [13, 42].

Many prior studies comparing healthy donors with cancer patients have focused mainly on standard immune cell types and maturation markers [43–48], while others [42, 49] have also examined several additional subsets. We report here that among the standard immune cell types, there were significantly higher levels of CD8^+^ T cells and lower levels of B cells in cancer patients compared to age-matched healthy donors (Figure 4). Notable differences in immune cells related to maturation and function were also evident between cancer patients and healthy donors. We show here for the first time that patients with multiple indications of advanced cancer had higher activation markers involved in immune checkpoint pathways in CD4^+^ and CD8^+^ T cells, several higher suppressive Treg and MDSC subsets, and altered expression of PD-L1 on APCs (Figure 5). CTLA-4 and PD-L1 are co-inhibitory molecules that are expressed on T cells upon activation [42, 50] and BATF is a transcription factor involved in PD-1 signalling [15]. Cancer patients had higher levels of BATF^+^CD4^+^ T cells, PD-L1^+^CD8^+^ T cells and CTLA-4^+^CD8^+^ T cells compared to healthy donors. Expression of PD-L1 on APCs was also altered in cancer patients, with lower levels detected on B cells and higher levels on pDCs when compared to healthy individuals.

Prior studies [51, 52] have shown that cancer patients also had higher levels of Tregs and MDSCs compared to healthy donors. In the studies reported here, while levels of total Tregs were similar to healthy donors, cancer patients had increased levels of CTLA-4^+^ Tregs, which is a phenotype of a biologically suppressive Treg [17]. It has also been previously reported that while prostate cancer patients had a similar frequency of total Tregs in peripheral blood as healthy donors, the CTLA-4^+^ Tregs from patients had greater suppressive functionality [53]. Additionally, patients with head and neck squamous cell carcinomas have also been shown to have increased levels of the CTLA-4^+^ on Tregs when compared to healthy donors [54]. While we did not find an increase in MDSCs in cancer patients in this study, cancer patients displayed an increased frequency in several subsets of MDSCs with a suppressive phenotype, including PD-L1^+^ MDSCs and gMDSCs.

In order to corroborate changes between cancer patients and healthy donors at the phenotypic level with those at the gene level, we performed genome-wide gene expression profiling in an additional subset of advanced cancer patients and age-matched healthy donors. In the flow cytometry analysis, some subsets of MDSCs were increased, while B cells were decreased in cancer patients relative to healthy donors. In the microarray data set, we observed a down-regulation of the MDSC suppressor CD200 and up-regulation of the MDSC activator CD300LB, as well as decreased expression of CD19 and several other markers of B cell maturation and activation. It has already been well-established that the identification of gene transcripts in PBMCs correlate to changes in survival, disease progression and other various clinical outcomes, especially in cancer [55–60]; however, these data demonstrate the potential for combining extensive phenotypical analysis with genome-wide gene expression analysis.

While the study reported here identified 123 immune cell subsets using flow cytometry, additional subsets could provide important insight regarding the effect of age and cancer on the immune system. For example, up to 17 unique B cell subsets have been described in the peripheral blood [61]; however, due to limitations in both the quantity of patient blood available, as well as the number of colours able to be run per panel on the LSR II, we were able to include only five B cell subsets in our study. Future studies might incorporate additional panels focusing on specific subsets such as B cells and NK cells – where additional phenotypic markers have been described in the literature – in order to gain deeper insight into the standard subsets. In addition, the utilization of new technologies such as CyTOF, the next generation of cell detection that allows for the simultaneous detection of up to 50 antibodies in a single panel, may overcome these limitations and allow for the efficient detection of numerous immune subsets from small samples [62, 63].

Analyses of the 123 immune cell subsets reported here required only 1 × 10^7^ PBMCs obtained from ∼5–10 mL of peripheral blood. This is a minimally invasive procedure and analyses could be performed immediately prior to immunotherapy and/or early in the agent regimen. Analyses of PBMC subsets can also identify changes in the patient's immune constitution as a result of prior and/or more recent therapies. Studies [64] have shown that some so-called “non-immune”-based therapies, such as certain chemotherapeutics and targeted therapies, can have appreciable effects (either positive or negative) on subsequent immunotherapy regimens. Recent hypothesis-generating clinical studies have also provided evidence in different immunotherapy trials that analyses of immune cell subsets may correlate with patient benefits [8, 42, 65]. Larger trials will need to be carried out to expand and confirm these findings. Purified PBMCs in this study were frozen and stored for batch analyses to reduce any assay to assay variations.

The studies reported here are meant to provide a foundation for the use of the 123 peripheral immune cell subset panels in future randomized clinical studies involving well-defined and homogeneous patient populations, as well as for use as an adjunct to other prognostic analyses such as the interrogation of patient biopsies, when available. In addition, to more carefully assess the changes in immune cell subsets as cancer progresses, longitudinal studies are planned that will include patients with different stages of cancer. Using these panels, we were able to identify differences in peripheral immune cell subsets between younger and older healthy donors, and between advanced cancer patients and age-matched controls. While the studies here focused on differences in age and between healthy donors and cancer patients, the analysis of 123 peripheral immune cell subsets can potentially also be employed to provide valuable information in the prognosis and/or therapy of patients with autoimmunity and other chronic or infectious diseases.

## 5. Compliance with Ethical Research Standards

All of the subjects gave informed consent. Samples from healthy donors were obtained from the NIH Clinical Center Blood Bank (NCT00001846), and patients were enrolled in clinical trials approved by the NCI IRB (NCT01772004, NCT00088413).

## References

[1] Whiteside TL Immune suppression in cancer: Effects on immune cells, mechanisms and future therapeutic intervention. Semin Cancer Biol. 2006;16:3–15. DOI:10.1016/j.semcancer.2005.07.008 16153857 10.1016/j.semcancer.2005.07.008

[2] Desai A Grolleau-Julius A Yung R Leukocyte function in the aging immune system. J Leukoc Biol. 2010;87:1001–9. DOI:10.1189/jlb.0809542 20200405 10.1189/jlb.0809542 PMC4057658

[3] Zanussi S Serraino D Dolcetti R Berretta M De Paoli P Cancer, aging and immune reconstitution. Anticancer Agents Med Chem. 2013;13:1310–24. 24102279 10.2174/18715206113136660348

[4] Chattopadhyay PK Roederer M Cytometry: Today's technology and tomorrow's horizons. Methods. 2012;57:251–8. DOI:10.1016/j.ymeth. 2012.02.009 22391486 10.1016/j.ymeth.2012.02.009 PMC3374038

[5] Perfetto SP Chattopadhyay PK Roederer M Seventeen-colour flow cytometry: Unravelling the immune system. Nat Rev Immunol. 2004;4:648–55. DOI:10.1038/nri1416 15286731 10.1038/nri1416

[6] Camous X Pera A Solana R Larbi A NK cells in healthy aging and age-associated diseases. J Biomed Biotechnol. 2012; 2012: 195956. DOI:10.1155/2012/195956 23251076 10.1155/2012/195956 PMC3517269

[7] Howlader N Na Krapcho M Garshell J Miller D Altekruse SF Kosary CL Yu M Ruhl J Tatalovich Z Mariotto A Lewis DR Chen HS Feuer EJ Cronin KA, (eds). SEER Cancer Statistics Review 1975–2012. National Cancer Institute; 2015; Available from: http://seer.cancer.gov/csr/1975_2012/ [Accessed: 2015-10-5].

[8] Jochems C Tucker JA Tsang KY Madan RA Dahut WL Liewehr DJ Steinberg SM Gulley JL Schlom J A combination trial of vaccine plus ipilimumab in metastatic castration-resistant prostate cancer patients: Immune correlates. Cancer Immunol Immunother. 2014; 63: 407–18. DOI: 10.1007/s00262-014-1524-0 24514956 10.1007/s00262-014-1524-0 PMC6314199

[9] Madan RA Bilusic M Heery C Schlom J Gulley JL Clinical evaluation of TRICOM vector therapeutic cancer vaccines. Semin Oncol. 2012; 39: 296–304. Epub 2012/05/19. DOI:10.1053/j.seminoncol. 2012. 02.010 22595052 10.1053/j.seminoncol.2012.02.010 PMC3398615

[10] Boyerinas B Jochems C Fantini M Heery CR Gulley JL Tsang KY Schlom J Antibody-Dependent Cellular Cytotoxicity Activity of a Novel Anti-PD-L1 Antibody Avelumab (MSB0010718C) on Human Tumor Cells. Cancer Immunol Res. 2015. DOI:10.1158/2326-6066.CIR-15-0059 10.1158/2326-6066.CIR-15-0059 PMC4739754 26014098

[11] Idorn M Kollgaard T Kongsted P Sengelov L Straten P Thor Correlation between frequencies of blood monocytic myeloid-derived suppressor cells, regulatory T cells and negative prognostic markers in patients with castration-resistant metastatic prostate cancer. Cancer Immunol Immunother. 2014;63:1177–87. DOI:10.1007/s00262-014-1591-2 25085000 10.1007/s00262-014-1591-2 PMC11028426

[12] Feise RJ Do multiple outcome measures require p-value adjustment? BMC Med Res Methodol. 2002;2:8. 12069695 10.1186/1471-2288-2-8 PMC117123

[13] Baitsch L Legat A Barba L Marraco SA Fuertes Rivals JP Baumgaertner P Christiansen-Jucht C Bouzourene H Rimoldi D Pircher H Rufer N Matter M Michielin O Speiser DE Extended co-expression of inhibitory receptors by human CD8 T-cells depending on differentiation, antigen-specificity and anatomical localization. PLoS One. 2012;7:e30852. DOI:10.1371/journal.pone.0030852 22347406 10.1371/journal.pone.0030852 PMC3275569

[14] Foks AC Ran IA Wasserman L Frodermann V Ter Borg MN de Jager SC van Santbrink PJ Yagita H Akiba H Bot I Kuiper J van Puijvelde GH T-cell immunoglobulin and mucin domain 3 acts as a negative regulator of atherosclerosis. Arterioscler Thromb Vasc Biol. 2013;33:2558–65. DOI:10.1161/ATVBAHA.113.301879 23990206 10.1161/ATVBAHA.113.301879

[15] Quigley M Pereyra F Nilsson B Porichis F Fonseca C Eichbaum Q Julg B Jesneck JL Brosnahan K Imam S Russell K Toth I Piechocka-Trocha A Dolfi D Angelosanto J Crawford A Shin H Kwon DS Zupkosky J Francisco L Freeman GJ Wherry EJ Kaufmann DE Walker BD Ebert B Haining WN Transcriptional analysis of HIV-specific CD8+ T cells shows that PD-1 inhibits T cell function by upregulating BATF. Nat Med. 2010;16:1147–51. DOI:10.1038/nm.2232 20890291 10.1038/nm.2232 PMC3326577

[16] D'Cruz LM Rubinstein MP Goldrath AW Surviving the crash: Transitioning from effector to memory CD8+ T cell. Semin Immunol. 2009;21:92–8. DOI: 10.1016/j.smim.2009.02.002 19269192 10.1016/j.smim.2009.02.002 PMC2671236

[17] Sansom DM Walker LS The role of CD28 and cytotoxic T-lymphocyte antigen-4 (CTLA-4) in regulatory T-cell biology. Immunol Rev. 2006; 212:131–48. DOI: 10.1111/j.0105-2896. 2006.00419.x 16903911 10.1111/j.0105-2896.2006.00419.x

[18] Noman MZ Desantis G Janji B Hasmim M Karray S Dessen P Bronte V Chouaib S PD-L1 is a novel direct target of HIF-1alpha, and its blockade under hypoxia enhanced MDSC-mediated T cell activation. J Exp Med. 2014;211:781–90. DOI:10.1084/jem.20131916 24778419 10.1084/jem.20131916 PMC4010891

[19] Duffy A Zhao F Haile L Gamrekelashvili J Fioravanti S Ma C Kapanadze T Compton K Figg WD Greten TF Comparative analysis of monocytic and granulocytic myeloid-derived suppressor cell subsets in patients with gastrointestinal malignancies. Cancer Immunol Immunother. 2013;62:299–307. DOI:10.1007/s00262-012-1332-3 23011590 10.1007/s00262-012-1332-3 PMC6628699

[20] Ma Y Shurin GV Gutkin DW Shurin MR Tumor associated regulatory dendritic cells. Semin Cancer Biol. 2012;22:298–306. DOI:10.1016/j.semcancer. 2012.02.010 22414911 10.1016/j.semcancer.2012.02.010 PMC3373995

[21] Thibult ML Mamessier E Gertner-Dardenne J Pastor S Just-Landi S Xerri L Chetaille B Olive D PD-1 is a novel regulator of human B-cell activation. Int Immunol. 2013;25:129–37. DOI:10.1093/intimm/dxs098 23087177 10.1093/intimm/dxs098

[22] Barclay AN Wright GJ Brooke G Brown MH CD200 and membrane protein interactions in the control of myeloid cells. Trends Immunol. 2002;23:285–90. Epub 2002/06/20. 12072366 10.1016/s1471-4906(02)02223-8

[23] Martinez-Barriocanal A Sayos J Molecular and functional characterization of CD300b, a new activating immunoglobulin receptor able to transduce signals through two different pathways. J Immunol. 2006;177:2819–30. Epub 2006/08/22. 16920917 10.4049/jimmunol.177.5.2819

[24] Tedder TF Poe JC Fujimoto M Haas KM Sato S The CD19-CD21 signal transduction complex of B lymphocytes regulates the balance between health and autoimmune disease: Systemic sclerosis as a model system. Curr Dir Autoimmun. 2005;8:55–90. Epub 2004/11/27. DOI:10.1159/000082087 15564717 10.1159/000082087

[25] Tedder TF Tuscano J Sato S Kehrl JH CD22, a B lymphocyte-specific adhesion molecule that regulates antigen receptor signaling. Annu Rev Immunol. 1997;15:481–504. Epub 1997/01/01. DOI:10.1146/annurev.immunol.15.1.481 9143697 10.1146/annurev.immunol.15.1.481

[26] Yamazaki T Nagumo H Hayashi T Sugane K Agematsu K CD72-mediated suppression of human naive B cell differentiation by down-regulating X-box binding protein 1. Eur J Immunol. 2005;35:2325–34. Epub 2005/07/28. DOI:10.1002/eji.200425639 16047337 10.1002/eji.200425639

[27] Schmitt C Tonnelle C Dalloul A Chabannon C Debre P Rebollo A Aiolos and Ikaros: Regulators of lymphocyte development, homeostasis and lymphoproliferation. Apoptosis. 2002;7:277–84. Epub 2002/05/09. 11997672 10.1023/a:1015372322419

[28] Lu X Sicard R Jiang X Stockus JN McNamara G Abdulreda M Moy VT Landgraf R Lossos IS HGAL localization to cell membrane regulates B-cell receptor signaling. Blood. 2015;125:649–57. Epub 2014/11/09. DOI: 10.1182/ blood-2014-04-571331 25381061 10.1182/blood-2014-04-571331 PMC4304110

[29] Roederer M Tarnok A OMIPs – Orchestrating multiplexity in polychromatic science. Cytometry A. 2010;77:811–2. Epub 2010/08/20. DOI:10.1002/cyto.a.20959 20722007 10.1002/cyto.a.20959

[30] Brinkman RR Aghaeepour N Finak G Gottardo R Mosmann T Scheuermann RH State-of-the-Art in the Computational Analysis of Cytometry Data. Cytometry A. 2015;87:591–3. Epub 2015/06/26. DOI: 10.1002/cyto.a.22707 26111230 10.1002/cyto.a.22707

[31] Robinson JP Rajwa B Patsekin V Davisson VJ Computational analysis of high-throughput flow cytometry data. Expert Opin Drug Discov. 2012;7:679–93. Epub 2012/06/20. DOI:10.1517/17460441.2012.693475 22708834 10.1517/17460441.2012.693475 PMC4389283

[32] Castelo-Branco C Soveral I The immune system and aging: A review. Gynecol Endocrinol. 2014;30:16–22. DOI:10.3109/09513590.2013.852531 24219599 10.3109/09513590.2013.852531

[33] Ligthart GJ Corberand JX Fournier C Galanaud P Hijmans W Kennes B Muller-Hermelink HK Steinmann GG Admission criteria for immunogerontological studies in man: The SENIEUR protocol. Mech Ageing Dev. 1984;28:47–55. 6513613 10.1016/0047-6374(84)90152-0

[34] Plackett TP Boehmer ED Faunce DE Kovacs EJ Aging and innate immune cells. J Leukoc Biol. 2004;76:291–9. DOI:10.1189/jlb.1103592 15039467 10.1189/jlb.1103592

[35] Czesnikiewicz-Guzik M Lee WW Cui D Hiruma Y Lamar DL Yang ZZ Ouslander JG Weyand CM Goronzy JJ T cell subset-specific susceptibility to aging. Clin Immunol. 2008;127:107–18. DOI:10.1016/j.clim.2007.12.002 18222733 10.1016/j.clim.2007.12.002 PMC2435295

[36] Gregg R Smith CM Clark FJ Dunnion D Khan N Chakraverty R Nayak L Moss PA The number of human peripheral blood CD4+ CD25high regulatory T cells increases with age. Clin Exp Immunol. 2005; 140: 540–6. DOI: 10.1111/j. 1365-2249. 2005.02798.x 15932517 10.1111/j.1365-2249.2005.02798.x PMC1809384

[37] Verschoor CP Johnstone J Millar J Dorrington MG Habibagahi M Lelic A Loeb M Bramson JL Bowdish DM Blood CD33(+)HLA-DR(−) myeloid-derived suppressor cells are increased with age and a history of cancer. J Leukoc Biol. 2013; 93:633–7. DOI: 10.1189/jlb. 0912461 23341539 10.1189/jlb.0912461 PMC3701116

[38] Borrego F Alonso MC Galiani MD Carracedo J Ramirez R Ostos B Pena J Solana R NK phenotypic markers and IL2 response in NK cells from elderly people. Exp Gerontol. 1999;34:253–65. 10363791 10.1016/s0531-5565(98)00076-x

[39] Gayoso I Sanchez-Correa B Campos C Alonso C Pera A Casado JG Morgado S Tarazona R Solana R Immunosenescence of human natural killer cells. J Innate Immun. 2011;3:337–43. DOI: 10.1159/000328005 21576928 10.1159/000328005

[40] Le Garff-Tavernier M Beziat V Decocq J Siguret V Gandjbakhch F Pautas E Debre P Merle-Beral H Vieillard V Human NK cells display major phenotypic and functional changes over the life span. Aging Cell. 2010;9:527–35. DOI: 10.1111/j. 1474-9726. 2010. 00584.x 20477761 10.1111/j.1474-9726.2010.00584.x

[41] Pawelec G Hirokawa K Fulop T Altered T cell signalling in ageing. Mech Ageing Dev. 2001;122:1613–37. 11511400 10.1016/s0047-6374(01)00290-1

[42] Santegoets SJ Stam AG Lougheed SM Gall H Scholten PE Reijm M Jooss K Sacks N Hege K Lowy I Cuillerot JM von Blomberg BM Scheper RJ van den Eertwegh AJ Gerritsen WR de Gruijl TD T cell profiling reveals high CD4+CTLA-4 + T cell frequency as dominant predictor for survival after prostate GVAX/ipilimumab treatment. Cancer Immunol Immunother. 2013;62:245–56. DOI: 10.1007/s00262-012-1330-5 22878899 10.1007/s00262-012-1330-5 PMC11029684

[43] Caras I Grigorescu A Stavaru C Radu DL Mogos I Szegli G Salageanu A Evidence for immune defects in breast and lung cancer patients. Cancer Immunol Immunother. 2004;53:1146–52. DOI: 10.1007/s00262-004-0556-2 15185014 10.1007/s00262-004-0556-2 PMC11034324

[44] Evans CF Galustian C Bodman-Smith M Dalgleish AG Kumar D The effect of colorectal cancer upon host peripheral immune cell function. Colorectal Dis. 2010; 12: 561–9. DOI: 10.1111/j. 1463-1318.2009.01819.x 19250260 10.1111/j.1463-1318.2009.01819.x

[45] Heuvers ME Muskens F Bezemer K Lambers M Dingemans AM Groen HJ Smit EF Hoogsteden HC Hegmans JP Aerts JG Arginase-1 mRNA expression correlates with myeloid-derived suppressor cell levels in peripheral blood of NSCLC patients. Lung Cancer. 2013;81:468–74. DOI:10.1016/j.lungcan.2013.06.005 23850196 10.1016/j.lungcan.2013.06.005

[46] Melichar B Touskova M Solichova D Kralickova P Kopecky O CD4(+) T-lymphocytopenia and systemic immune activation in patients with primary and secondary liver tumours. Scandinavian Journal of Clinical & Laboratory Investigation. 2001;61:363–70. DOI: 10.1080/003655101316911404 11569483 10.1080/003655101316911404

[47] Wang L Chang EW Wong SC Ong SM Chong DQ Ling KL Increased myeloid-derived suppressor cells in gastric cancer correlate with cancer stage and plasma S100A8/A9 proinflammatory proteins. J Immunol. 2013;190:794–804. DOI:10.4049/jimmunol.1202088 23248262 10.4049/jimmunol.1202088

[48] Yamamoto T Yanagimoto H Satoi S Toyokawa H Yamao J Kim S Terakawa N Takahashi K Kwon AH Circulating myeloid dendritic cells as prognostic factors in patients with pancreatic cancer who have undergone surgical resection. J Surg Res. 2012;173:299–308. DOI:10.1016/j.jss.2010.09.027 21195425 10.1016/j.jss.2010.09.027

[49] MacFarlane AW Jillab M Plimack ER Hudes GR Uzzo RG Litwin S Dulaimi E Al-Saleem T Campbell KS PD-1 Expression on Peripheral Blood Cells Increases with Stage in Renal Cell Carcinoma Patients and Is Rapidly Reduced after Surgical Tumor Resection. Cancer Immunol Res. 2014;2:320–31. DOI:10.1158/2326-6066.CIR-13-0133 24764579 10.1158/2326-6066.CIR-13-0133 PMC4007343

[50] Keir ME Butte MJ Freeman GJ Sharpe AH PD-1 and its ligands in tolerance and immunity. Annu Rev Immunol. 2008;26:677–704. DOI:10.1146/annurev.immunol.26.021607.090331 18173375 10.1146/annurev.immunol.26.021607.090331 PMC10637733

[51] Elkord E Alcantar-Orozco EM Dovedi SJ Tran DQ Hawkins RE Gilham DE T regulatory cells in cancer: Recent advances and therapeutic potential. Expert Opin Biol Ther. 2010;10:1573–86. DOI:10.1517/14712598.2010.529126 20955112 10.1517/14712598.2010.529126

[52] Solito S Marigo I Pinton L Damuzzo V Mandruzzato S Bronte V Myeloid-derived suppressor cell heterogeneity in human cancers. Ann N Y Acad Sci. 2014;1319:47–65. DOI:10.1111/nyas.12469 24965257 10.1111/nyas.12469

[53] Yokokawa J Cereda V Remondo C Gulley JL Arlen PM Schlom J Tsang KY Enhanced functionality of CD4+CD25(high)FoxP3+ regulatory T cells in the peripheral blood of patients with prostate cancer. Clin Cancer Res. 2008;14:1032–40. DOI: 10.1158/ 1078-0432. CCR-07-2056 18281535 10.1158/1078-0432.CCR-07-2056

[54] Strauss L Bergmann C Gooding W Johnson JT Whiteside TL The frequency and suppressor function of CD4+CD25highFoxp3+ T cells in the circulation of patients with squamous cell carcinoma of the head and neck. Clin Cancer Res. 2007;13:6301–11. DOI: 10.1158/1078-0432.CCR-07-1403 17975141 10.1158/1078-0432.CCR-07-1403

[55] Baine MJ Chakraborty S Smith LM Mallya K Sasson AR Brand RE Batra SK Transcriptional profiling of peripheral blood mononuclear cells in pancreatic cancer patients identifies novel genes with potential diagnostic utility. PLoS One. 2011;6:e17014. Epub 2011/02/25. DOI:10.1371/journal.pone.0017014 21347333 10.1371/journal.pone.0017014 PMC3037404

[56] Kiaii S Clear AJ Ramsay AG Davies D Sangaralingam A Lee A Calaminici M Neuberg DS Gribben JG Follicular lymphoma cells induce changes in T-cell gene expression and function: Potential impact on survival and risk of transformation. J Clin Oncol. 2013;31:2654–61. Epub 2013/06/19. DOI:10.1200/JCO.2012.44.2137 23775959 10.1200/JCO.2012.44.2137 PMC3709054

[57] Herazo-Maya JD Noth I Duncan SR Kim S Ma SF Tseng GC Feingold E Juan-Guardela BM Richards TJ Lussier Y Huang Y Vij R Lindell KO Xue J Gibson KF Shapiro SD Garcia JG Kaminski N Peripheral blood mononuclear cell gene expression profiles predict poor outcome in idiopathic pulmonary fibrosis. Sci Transl Med. 2013;5:205ra136. Epub 2013/10/04. DOI:10.1126/scitranslmed.3005964 10.1126/scitranslmed.3005964 PMC4175518 24089408

[58] Sitras V Fenton C Acharya G Gene expression profile in cardiovascular disease and preeclampsia: A meta-analysis of the transcriptome based on raw data from human studies deposited in Gene Expression Omnibus. Placenta. 2015;36:170–8. Epub 2015/01/04. DOI:10.1016/j.placenta.2014.11.017 25555499 10.1016/j.placenta.2014.11.017

[59] Ma J Lin Y Zhan M Mann DL Stass SA Jiang F Differential miRNA expressions in peripheral blood mononuclear cells for diagnosis of lung cancer. Lab Invest. 2015;95:1197–206. Epub 2015/07/07. DOI:10.1038/labinvest.2015.88 26146958 10.1038/labinvest.2015.88 PMC4586315

[60] Komatsu N Matsueda S Tashiro K Ioji T Shichijo S Noguchi M Yamada A Doi A Suekane S Moriya F Matsuoka K Kuhara S Itoh K Sasada T Gene expression profiles in peripheral blood as a biomarker in cancer patients receiving peptide vaccination. Cancer. 2012;118:3208–21. Epub 2011/11/11. DOI:10.1002/cncr.26636 22071976 10.1002/cncr.26636

[61] Qian Y Wei C Lee F Eun-Hyung Campbell J Halliley J Lee JA Cai J Kong YM Sadat E Thomson E Dunn P Seegmiller AC Karandikar NJ Tipton CM Mosmann T Sanz I Scheuermann RH Elucidation of seventeen human peripheral blood B-cell subsets and quantification of the tetanus response using a density-based method for the automated identification of cell populations in multidimensional flow cytometry data. Cytometry B Clin Cytom. 2010;78 Suppl 1:S69–82. Epub 2010/09/21. DOI:10.1002/cyto.b.20554 20839340 10.1002/cyto.b.20554 PMC3084630

[62] Cheung RK Utz PJ Screening: CyTOF-the next generation of cell detection. Nat Rev Rheumatol. 2011;7:502–3. Epub 2011/07/27. DOI: 10.1038/nrrheum. 2011.110 21788983 10.1038/nrrheum.2011.110 PMC3387986

[63] Yao Y Liu R Shin MS Trentalange M Allore H Nassar A Kang I Pober JS Montgomery RR CyTOF supports efficient detection of immune cell subsets from small samples. J Immunol Methods. 2014;415:1–5. Epub 2014/12/03. DOI:10.1016/j.jim.2014.10.010 25450003 10.1016/j.jim.2014.10.010 PMC4269324

[64] Roselli M Cereda V di Bari MG Formica V Spila A Jochems C Farsaci B Donahue R Gulley JL Schlom J Guadagni F Effects of conventional therapeutic interventions on the number and function of regulatory T cells. Oncoimmunology. 2013;2:e27025. DOI:10.4161/onci.27025 24353914 10.4161/onci.27025 PMC3862634

[65] Santegoets SJ Stam AG Lougheed SM Gall H Jooss K Sacks N Hege K Lowy I Scheper RJ Gerritsen WR van den Eertwegh AJ de Gruijl TD Myeloid derived suppressor and dendritic cell subsets are related to clinical outcome in prostate cancer patients treated with prostate GVAX and ipilimumab. J Immunother Cancer. 2014;2:31. DOI:10.1186/s40425-014-0031-3 26196012 10.1186/s40425-014-0031-3 PMC4507359

